# Smoking prevalence in the area of responsibility of the German Ministry of Defense — results of the employee survey in the context of occupational health management

**DOI:** 10.1186/s40779-020-00236-w

**Published:** 2020-02-21

**Authors:** Stefan Sammito, Annika Claus, Dirk-Matthias Rose

**Affiliations:** 1 Department I 3 Research & Development, Air Force Centre of Aerospace Medicine, Cologne, Germany; 2 Occupational Medicine, Faculty of Medicine, Otto-von-Guericke-University of Magdeburg, Magdeburg, Germany; 3 Institute for Teachers’ Health, Institute of Occupational, Social and Environmental Medicine of the Johannes-Gutenberg University Mainz, Mainz, Germany

**Keywords:** Prevention, Smoking, Military, Health promotion

## Abstract

**Background:**

Tobacco use, correlated with reduced physical fitness, is one of the leading causes of avoidable death worldwide. It increases the risk of dementia and can shorten the lifespan by 10 years. For the German Armed Forces (Bundeswehr), figures on smoking behavior have not been comprehensively captured. This study analyzes current data in a large sample from this population.

**Methods:**

Based on an employee survey as part of the Occupational Health Management System, data on smoking behavior from 13,326 participants were analyzed in relation to age, gender, professional status, education level and membership in military operational units versus other agencies.

**Results:**

Smoking behavior varied significantly (*P <* 0.001) by age group (younger > older), gender, professional status, military agency membership status, and education level (the lower the education level, the higher the smoking rates). With the exception of the downward trend in smoking behavior with increasing age among civilian employees, these results were all significant (*P <* 0.005).

**Conclusions:**

This data analysis shows that smoking prevalence among personnel in the area of responsibility of the Federal Ministry of Defense is comparable to the current data from corresponding surveys of the German population. Depending on gender, they generally show values that are slightly above those of the German population. The well-known trend in the general population of decreasing smoking prevalence with increasing age is also seen in this analysis. However, there are considerable differences in the smoking prevalence among individual subgroups (professional status, agency, gender, education level). The data show that particular young soldiers in the armed forces should be the target group for further preventive measures.

## Background

Tobacco use is one of the leading causes of avoidable death worldwide [1]. Smoking affects human health, as heart attacks, stroke, arteriosclerosis, pneumonia, chronic bronchitis, and malignant neoplasm of the lung, oral cavity, larynx and digestive organs [2] are more likely to occur in smokers. Smoking is still the most important risk factor for cancer and responsible for more than 85,000 cases of cancer (more than 50% of them are lung cancer cases) [3]. Active smoking also increases the risk of dementia in middle-aged groups [4]. In addition, chronic inflammation from exposure to cigarette smoke increases when combined with other inhaled toxicants [5]. On average, smokers die 10 years earlier than nonsmokers [6].

**Fig. 1:**
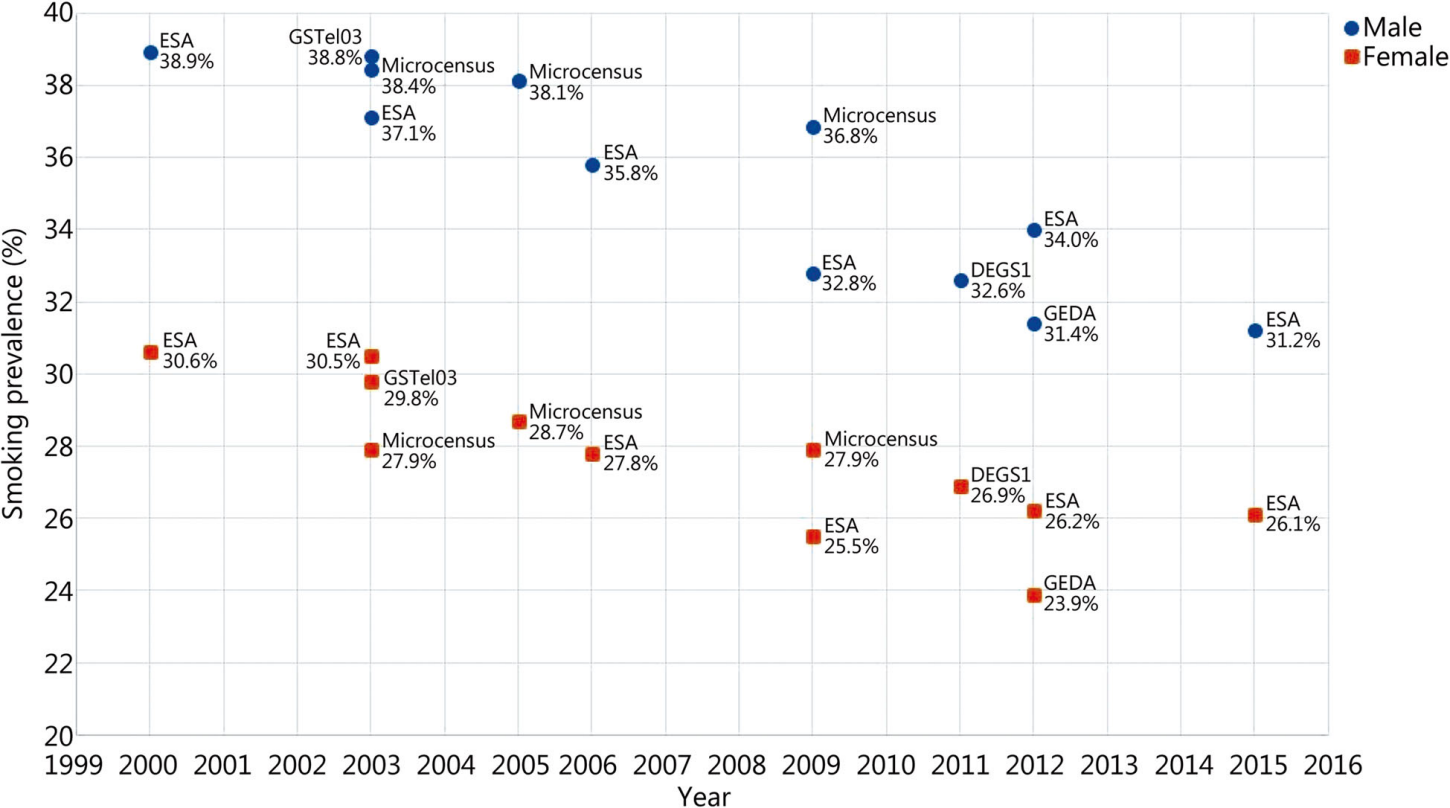
Smoking prevalence in the German population of working age. ESA: Epidemiological addiction survey; DEGS: German Health Interview and Examination Survey for Adults; GEDA: German Health Update; GSTel03: Telephone Health Survey; Data taken from reference [7–18]

Despite a general downward trend in the past 20 years, 26–31% of women and 31–39% of men in Germany are smokers, depending on the study [7–18] (Fig. 1).

Smoking is also significantly correlated with reduced physical fitness [19, 20]. Against this background, the sustainable reduction of tobacco use and protection from passive smoking are the objectives of Health and Prevention Policy [21, 22] and promoted in the context of addiction prevention as part of Occupational Health Management (OHM) measures in the operational environment [23, 24]. A clinical guideline on “Screening, Diagnostics and Treatment of Harmful and Addictive Tobacco Use” [25] assists physicians and provides advice to individuals regarding the scientific evidence of suitable measures.

For the area of the Bundeswehr, the available figures on smoking behavior among soldiers^1^ are limited and not up to date [26–29]. While environmental preventive measures (e.g., smoke-free premises) have been established for years, behavioral preventive measures for reducing tobacco use among active employees of the Federal Ministry of Defense (FMoD) are available only to a limited extent. These preventive measures are part of the medical care and limited to rehabilitation or addiction therapy. As part of the introduction of OHM for the area of responsibility of the FMoD, the scientific treatment of smoking prevalence with the aim of identifying suitable target groups for addiction prevention has shown a considerable increase in importance.

The aim of this study is to quantify the number of active employees in the area of responsibility of the German Ministry of Defense based on the results of an employee survey in the context of the OHM system. Furthermore, the percentage of active smokers should be analyzed with regard to age, status (military vs. civilian), gender and education level.

## Methods

As part of the introduction of OHM, Bundeswehr agencies have been interviewed systematically since May 2017 via the AIGScreenBw^2^ employee survey. This data analysis is based on a survey of 119 agencies with a total of 63,292 employees. Of these, 39,973 are military personnel and 23,319 are civilian personnel.

The employee survey was performed both as a purely online survey and a combined online/paper-pencil survey. The latter could be requested by the agencies if not all employees had Internet access at the office.

The number of smokers is calculated from all subjects who gave a specific figure in response to the (open) question “How many cigarettes/ cigars/ pipes do you consume every day on average?” Nonsmokers were requested to enter “0”. Incomplete information was excluded from further analysis. Furthermore, smoking behavior in relation to the categorized age (“How old are you?” with possible responses of “younger than 30 years”, “30–39 years”, “40–49 years” and “50 years and older”), status group (“soldier”, “civil servant” and “salaried employee”) and last school-leaving qualification (“What is your highest level of education?” with possible responses of “no schooling”, “lower school-leaving certificate”, “intermediate school-leaving certificate”, “higher education entrance qualification”, and “other school-leaving qualification”) was analyzed. All agencies surveyed were categorized into task forces (primarily military agencies with direct operational relevance) or other agencies (including military staff and military medical facilities).

In addition to descriptive statistics with the percentage rate of active smokers, the differences were calculated by applying the chi-square test using IBM® SPSS® Statistics 24 (IBM®, Armonk, NY, USA). To adjust for multiple comparisons, a Bonferroni correction was used. A corrected *P <* 0.002 (0.05/31) was considered statistically significant and was further interpreted.

For this survey, there is a positive vote by the ethics panel of the Medical Association Rhineland-Palatinate (No. 837.503.17 [11337]). The processing of the research issue is performed as part of a collaborative research project (registration number 06KS-S-631619) and a research assignment (assignment number E/U2AD/HD003/HD001), and was approved by the FMoD under registration numbers 3/01/17 and 3/04/18.

## Results

Altogether, 16,798 employees participated in the survey, which corresponds to a participation rate of 26.6%. The average agency-related response rate was 34.0% (range: minimum 3.8%, maximum 80.1%, standard deviation 17.7%). For the descriptive analysis, only cases with full details on smoking behavior and relevant sociodemographic characteristics (age, gender, education level) were used. In 3472 cases (20.7%), at least one of these details was missing, which is why they were excluded from the analysis.

Of the 13,326 individuals who participated in the survey, 9120 (68.4%) were soldiers and 4206 (31.6%) were civilians; 2956 were women (22.2%), and 10,370 were men (77.8%). The group of people under 30 was the largest group (28.5%), with 3799 participants. The distributions correspond to the population in terms of status, gender and age see Table 1.

**Table 1:** Comparison of the sample with the population of all potential participants in terms of status, gender and age

Index	Population [*n*(%)]	Sample[*n*(%)]	*P*
Status			0.91
Soldiers	39,973 (63.2)	9120 (68.4)	
Civilian employees	23,319 (36.8)	4206 (31.6)	
Gender			0.95
Male	50,683 (80.1)	10,370 (77.8)	
Female	12,607 (19.9)	2956 (22.2)	
Age			1.00
< 30 years	16,078 (25.4)	3799 (28.5)	
30–40 years	16,918 (26.7)	3707 (27.8)	
40–50 years	12,729 (20.1)	2725 (20.4)	
≥ 50 years	17,535 (27.1)	3095 (23.2)	

In the overall population, the smoking rate is 32.5%. There is a significant difference in the smoking rate with regard to gender (men: 34.1%, women: 27.0%; *P <* 0.001), status (soldiers: 37.1%, civilian personnel: 22.7%; *P <* 0.001), employment in a task force versus other agencies (task forces: 45.0%, all other agencies: 26.0%; *P <* 0.001) and education level (lower school-leaving certificate: 47.4%, intermediate school-leaving certificate 38.7%, higher education entrance qualification 23.6%, other: 30.3%; *P <* 0.001).

**Fig. 2:**
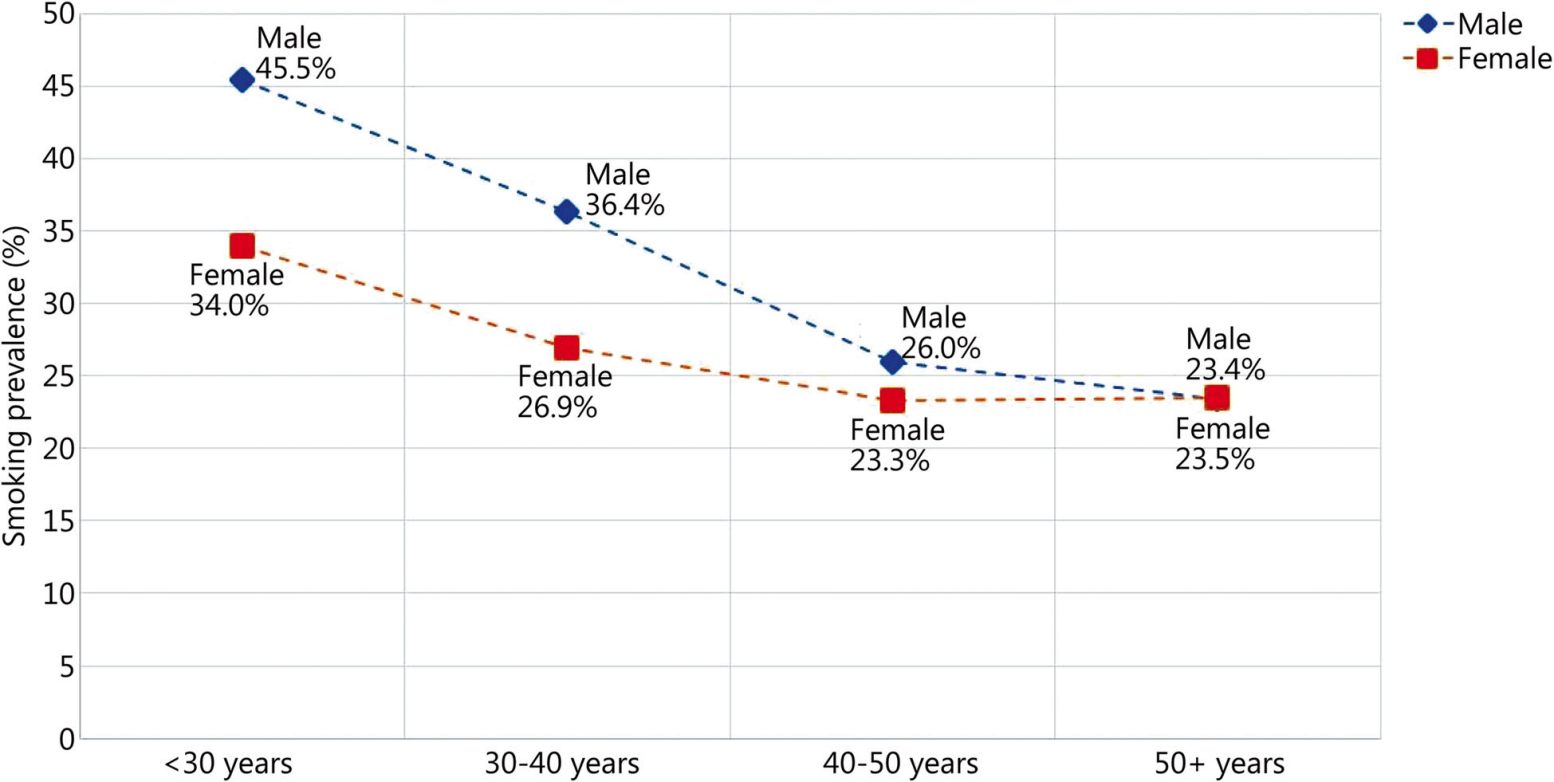
Trend of smoking prevalence in the population surveyed among employees in the area of responsibility of the Federal Ministry of Defense, separated by gender (*n* = 13,326)

In the analysis of the four age groups of the overall sample, there was a significant difference among the age groups, with the smoking rate of 43.3% among people under 30 decreasing to 23.5% among people over 50 (see Fig. 2). This age trend was also apparent in the relevant sub-analyses with regard to gender, status, employment in a task force and education level, with the differences among people aged 50 and older between men and women (23.4% or 23.6%, *P* = 0.963) and between military and civilian personnel (23.0% or 23.7%, *P* = 0.692). This trend across the different age groups was not found only among civilian personnel, and smoking prevalence remained nearly constant. An overview of the group differences is shown in Table 2.

**Table 2:** Smoking prevalence by agency, employee status, gender and education level

Only smoking rate	Total	Age [*n*(%)]				*P*
		< 30 years (*n* = 3799)	30–40 years (*n* = 3707)	40–50 year (*n* = 2725)	≥50 years (*n* = 3095)	
All (*n* = 13,326)	4336 (32.5)	1644 (43.3)	1270 (34.3)	696 (25.5)	726 (23.5)	*<* 0.001
Agency						
Task forces (*n* = 4575)	2061 (45.0)	1173 48.4)	679 45.4)	143 (32.8)	66 (30.6)	*<* 0.001
All other agencies (*n* = 8751)	2275 (26.0)	471 34.3)	591 26.7)	553 (24.2)	660 (22.9)	*<* 0.001
*P*	*<* 0.001	*<* 0.001	*<* 0.001	*<* 0.001	0.012	
Status						
Soldiers (*n* = 9120)	3381 (37.1)	1538 (45.8)	1089 (38.0)	491 (27.9)	263 2(3.0)	*<* 0.001
Civilian employees (*n* = 4206)	955 (22.7)	106 (23.9)	181 (21.5)	205 (21.2)	463 (23.7)	0.324
*P*	*<* 0.001	*<* 0.001	*<* 0.001	*<* 0.001	0.692	
Gender						
Men (*n* = 10,370)	3538 (34.1)	1397 (45.5)	1053 (36.3)	570 (26.1)	518 (23.4)	*<* 0.001
Women (*n* = 2956)	798 (27.0)	247 (34.0)	217 (26.8)	126 (23.5)	208 (23.6)	*<* 0.001
*P*	*<* 0.001	*<* 0.001	*<* 0.001	0.225	0.963	
Education level						
Lower school-leaving certificate (*n* = 1226)	581 (47.4)	247 (62.2)	142 (54.0)	63 (41.7)	129 (31.1)	*<* 0.001
Intermediate school-leaving certificate (*n* = 5767)	2230 (38.7)	923 (47.0)	663 (42.6)	315 (29.3)	329 (28.1)	*<* 0.001
Higher education entrance qualification (*n* = 5911)	1397 (23.6)	431 (32.3)	427 (24.3)	297 (21.0)	242 (17.2)	*<* 0.001
Other (*n* = 422)	128 (30.3)	43 (41.3)	38 (29.7)	21 (23.9)	26 (25.5)	0.031
*P*	*<* 0.001	*<* 0.001	*<* 0.001	*<* 0.001	*<* 0.001	

## Discussion

This data analysis shows that smoking prevalence among employees in the area of responsibility of the FMoD is comparable to the current data from corresponding surveys of the German population. Depending on gender, these employees generally show smoking prevalence values that are slightly above those of the German population. The well-known trend in the general population of decreasing smoking prevalence with increasing age is also seen in this analysis. However, there are considerable differences in smoking prevalence among individual subgroups.

It must be taken into account that, in this data analysis, the group of younger employees was larger than the group of older employees, which also corresponds to the general age structure, particularly in the armed forces. However, since the prevalence of smoking is higher among the younger age groups, the slightly increased smoking prevalence in the overall sample may be based on this age distribution. A comparison of these findings with those of the latest microcensus survey from 2017 shows that smoking prevalence also depends on age in the general population [16]. However, the smoking prevalence in the sample examined here is clearly above the smoking prevalence in the overall population. This trend is particularly strong among military personnel, whereas the age-related reduction is only minor among civilian personnel. We can therefore assume that the differences observed in the smoking prevalence between the civilian population and the sample examined here apply particularly to military personnel. This conclusion is supported by the fact that, across all age groups, the smoking prevalence among the so-called “task forces” is higher than that among all other agency types. There could be several possible reasons for this, e.g., lower education, compensation for the risky job, and peer group influence. Additionally, the discrepancy between smoking habits on site and the high level of physical fitness needed to perform that kind of job is conspicuous. Additionally, the extent to which a lower avoidance of health risk factors due to a job deemed risky is an explanation for smoking behavior cannot be answered from the available data. However, these findings are consistent with the results of other studies, according to which veterans were convinced that smoking was part of everyday military life [30].

The subgroup analyses with regard to gender (more men than women smoke) and education lelvel (the lower the education level, the higher the smoking rates) correspond to the results from other surveys for Germany [9, 16, 31]. This is a key finding, especially for determining the target groups for smoking prevention programs.

This data analysis is the first survey of this type and scope. Previous publications have mostly focused on particular groups of soldiers [26–29]. For example, Glaser [26] examined smoking behavior and the body mass index among applicants and active air transport officers over a period of 30 years, which also showed a downward trend, but 21.7% of the applicants (during the 1997–2006 period) and 20.7% of the active air transport officers (2004) were smokers. In a study involving 1570 temporary-career volunteer candidates at the Medical Clinic with Specialty Services, Detmold, as part of reenlistment examinations, 54.8% were active smokers, with an average age of 23.8 years [27]. Thus, the smoking prevalence rates in this young study group are significantly higher than the rates shown here. A similarly high smoking prevalence rate (56.4%) was found by Wesemann et al. [28] among 264 mechanized infantry soldiers, with the majority of them (135 out of 149 soldiers) being classified as heavy smokers (> 20 cigarettes/day). In a study involving 1483 soldiers returning from deployment in Afghanistan and 889 soldiers without operational experience, Trautmann et al. [29] found a similarly high rate of active smoking (55.1%).

Although this data analysis is the largest of its kind so far, the special framework conditions of the AIGScreenBw employee survey must be taken into account when interpreting the results. With a total of 119 agencies and an overall strength of 63,292 employees, the sample comprises approximately 1/4 of the entire body of personnel in the area of responsibility of the FMoD. Nevertheless, it is a random selection of agencies because it only included the agencies that had conducted a corresponding employee survey since May 2017. In particular, the results from Army combat forces (for example, it was not possible to survey mechanized infantry battalions or tank battalions) and many of the agencies surveyed were command authorities. Furthermore, participating in the employee survey and answering the questions are voluntary. The underlying participation rate of 26.6% for all agencies, or the average participation rate of 34.0% when individual agencies are analyzed, can be improved, as well as in comparison with that of other employee surveys [32–34] in the area of OHM. On the whole, however, the participation rate has increased compared to that of the test phase (2015: 22.4% on average out of 11 test agencies [32] versus 34.0% here), which indicates a growing acceptance of the AIGScreenBw employee survey. Since all agencies surveyed had the option to choose between a purely online survey and a combined online/ paper-pencil survey, all employees could participate in the relevant survey. The authors do not expect any effects from the bimodal procedure. Rather, a lower response rate and high selectivity of the participants would have been expected if no paper-pencil surveys had been offered.

Another limitation is that the question concerning smoking behavior did not inquire into recent trends, such as the use of e-cigarettes, in a differentiated way. This may have led to an underestimation of the smoking prevalence in the underlying sample because smokers of e-cigarettes possibly did not feel that the question applied to them. This must be taken into account in the future through a differentiated survey in AIGScreenBw.

Against the background of the numerous recommendations for “smoking cessation” to reduce secondary diseases, among others, based on the clinical guideline on “Screening, Diagnostics and Treatment of Harmful and Addictive Tobacco Use” [25] in the academic literature [23, 35] and the success of behavioral preventive measures from a socio-political point of view [36], the available data show that there is also a need for action in the area of responsibility of the FMoD. In particular, given the short- and long-term effects of active smoking on health and physical fitness and thus on the operational capability of soldiers, the effective prevention of smoking addiction is an important field, especially for the armed forces. In this context, operational measures such as those in the OHM provide an effective and inexpensive way to reduce smoking prevalence, which has also been shown in a Cochrane analysis with 57 studies [37].

## Conclusions

In summary, the smoking prevalence in the area of responsibility of the FMoD is comparable to corresponding data from the German population, but given the special tasks of the FMoD, greater efforts to achieve a sustainable reduction should be made from a preventive medical perspective. The data show that in particular, young soldiers in the armed forces should be the target group for further preventive measures.

## Data Availability

The data that support the findings of this study are available from the Federal Ministry of Defense, but restrictions apply to the availability of these data, which were used under license for the current study and are therefore not publicly available.

## Abbreviations

AIGScreenBw: Arbeitsplatzmerkmale und individuellen Gesundheitsverhalten Screening Instrument Bundeswehr (Job Characteristics and Individual Health Behavior Screening Instrument of the Bundeswehr)
DEGS: German Health Interview and Examination Survey for Adults
ESA: Epidemiological addiction survey
FMoD: Federal Ministry of Defense
GEDA: German Health Update
GSTel03: Telephone Health Survey
OHM: Occupational Health Management

## Footnotes

1 For better readability, only the masculine form is used in the following text; this form always includes female members of the armed forces

2 AIGScreenBw: Arbeitsplatzmerkmale und individuellen Gesundheitsverhalten Screening Instrument Bundeswehr (Job Characteristics and Individual Health Behavior Screening Instrument of the Bundeswehr)
